# Clinical Lectures on Surgery Delivered at the Hospital of La Charité, by Professor Velpeau

**Published:** 1841-10

**Authors:** 


### Art. XI.

*Leçons Orales de Clinique Chirurgicale faites à l'Hôpital de la Charité, par M. le Professeur Velpeau.* Recueillés et publiées par le Docteur P. Pavillon.—*Paris,* 1840. 8vo, pp. 565.

*Clinical Lectures on Surgery delivered at the Hospital of la Charité, by Professor Velpeau.* Collected and published by Dr. P. Pavillon. —*Paris,* 1840.

The success that attended the publication of the Lectures of M. Dupuytren has induced other students to communicate to the public the clinical remarks of their professors also. The present volume is an instance. The extensive professional erudition, the accurate powers of observation, and the vast opportunities M. Velpeau possesses, render any remarks made by him interesting to the profession; and we have been much gratified by the perusal of this volume, though occasionally inclined to smile at the little pompous preface with which many of the different articles commence, denouncing the inadequacy of previous knowledge and heralding the additional light the professor is about to throw on the subject. We must confess, however, that the author has in some of these made good his premises.

The articles in the present volume on the subject of diseases of the eyes occupy more than one half of its pages; but as we have so fully commented on this department of surgery in the late numbers of our Journal, we omit any notice of them here, and confine our attention to the other principal subjects treated of. From the different articles, often unnecessarily prolix, we shall endeavour to glean and lay before our readers the chief original or peculiar views of the professor.

*Hydrocele.* In treating this subject M. Velpeau divides hydrocele into acute and chronic. The acute form, "which is little spoken of by authors though of sufficiently frequent occurrence," is caused by the same accidents that produce orchitis, of which it is almost a constant accompaniment, more especially of that variety of orchitis arising from discharges from the urethra. M. Velpeau combats the opinion of M. Rochoux, who affirms that acute hydrocele forms almost the entire swelling and disease in "gonorrhœal orchitis." M. Velpeau affirms that usually only a fourth or sixth of the bulk of the tumour is caused by the effusion into the tunica vaginalis, and he considers that the soft spongy feel that the enlarged testicle communicates has been mistaken for the effused fluid. He gives rules for distinguishing the two varieties; but as we cannot commend them for clearness or precision, we shall pass them over. The treatment of acute hydrocele is the same as that for common orchitis, with addition of the evacuation of the fluid in the former, which M. Velpeau occasionally employs. It is chiefly on this account that it is important to detect the complication of this acute hydrocele in orchitis, the symptoms being much relieved by puncture of the tumour. M. Velpeau at first performed this operation merely with the view of detecting the presence of fluid; but finding always where fluid, in however small a quantity, escaped, that the patient was relieved, he adopted the puncture as a regular mode of practice in such cases, and with great benefit. The puncture is made with the point of the lancet, gives immediate relief, and accelerates the cure where only the smallest quantity of fluid (or even it is said none at all) escapes.

M. Velpeau considers at great length the causes and treatment of chronic hydrocele. He has observed in many cases small cysts developed in the tunica albuginea or on the surface of the testicle or epididymis. Sir B. Brodie had already described a species of encysted hydrocele developed between the visceral layer of the tunica vaginalis and the tunica albuginea. The observation of M. Velpeau confirms that of Sir B. Brodie, the only difference between them being that the cyst observed by the latter was large and single, and those by M. Velpeau small and more numerous.

For the radical cure of hydrocele, M. Velpeau considers none of the numerous methods that have been used by surgeons so fitting as that of injection of the cavity. He expresses his surprise that the surgeons of London, Berlin, and America should still perform dangerous, long, and bloody operations, instead of the one he is in the habit of using. We think that since the publication of Pott's excellent treatise on this disease few surgeons in this country have deserved this reproach of M. Velpeau. But he himself recommends incision of the sac, in cases where the contained fluid is thick, or where the coats of the tunica vaginalis are so hard, thick, and solid that injection of the sac will fail to cure the disease; and in cases where the sac has degenerated into a stony or osteo-calcareous shell, or into lardaceous matter, he recommends excision. In the radical cure of hydrocele M. Velpeau has been in the habit of using an injection of tincture of iodine instead of wine or other stimulants, and he considers it to have the following advantages: a small quantity only is required, half an ounce to an ounce; it produces no ill consequences even if allowed to remain in the sac; it does not cause any serious injury if injected into the cellular tissues; and lastly it is almost uniformly successful. The cure is also more prompt, being effected in eight, ten, or twenty days. If there be swelling of the testicle, this also is frequently cured by the injection; and in cases where the swelling appears uninfluenced by the operation, M. Velpeau has found that at the end of five or six months it has entirely disappeared. The proportion of iodine used by the professor is two or three drachms of the tincture to an ounce of water; he has even used the pure tincture without any bad consequences: he considers that the reason why M. Fricke of Hamburg has failed in his operations, is that he has not made use of a sufficiently strong injection. M. Velpeau, reasoning from the relief afforded to the cases of acute hydrocele by puncture of the tunica vaginalis, has employed this means also after injection with the iodine solution, and has found that the tumour has been thereby much more rapidly discussed. One of the most eminent hospital surgeons of London has for some time been in the habit of using the iodine solution in the radical cure of hydrocele, and he has the same opinions of its efficacy as M. Velpeau. It is necessary to prepare the solution of iodine with the hydriodate of potass. In congenital hydrocele M. Velpeau recommends the iodine injection, while at the same time pressure is made on the inguinal canal.

*Dislocations of the Shoulder.* Injuries of this kind have been considered for some time a subject of surgery perfectly well understood; but M. Velpeau maintains that many of the views already set forth are either erroneous or incomplete. Considering it impossible for a dislocation to take place downwards on the inferior costa of the scapula, he divides the luxations of the shoulder into those which take place in a direction anterior and internal, or posterior and external to the glenoid cavity.

The posterior and external dislocations comprise complete dislocations on the infra-spinous fossa of the scapula (of which too many cases are now recorded to leave any doubt of their occurrence), and incomplete dislocations in the same direction. This species of luxation has not before been described by any writer on surgery. M. Velpeau instances three cases which he has met with in practice in which he considers this dislocation had occurred. He defines incomplete dislocations to be those in which "the humerus is arrested by some point of its anatomical neck on the border of the glenoid cavity.......If by incomplete dislocations," he adds, "we understand the cartilaginous surface of the head of the humerus to have only half escaped from the glenoid cavity, there is no doubt that we cannot admit the existence of such cases." The following are the symptoms, as described by the professor, of an external and posterior incomplete dislocation: "The arm half an inch longer than that of the opposite side, fixed immoveably by the side of the chest; the elbow directed forwards and inwards; the head of the humerus causing no prominence under the coracoid process, but projecting about six to eight lines on the posterior or subspinous border of the acromion; the axilla free, and the pectoralis major as well as the internal portion of the deltoid much depressed." In the case in which these symptoms were recorded, the dislocation had existed for ten or eleven months, and it was afterwards reduced by M. Sedillot, who considered the luxation a complete one. M. Velpeau gives the following reasons for believing the dislocation incomplete:

"If the dislocation had been complete, the head of the humerus would have been found more than an inch behind the summit of the acromion, but it was only a few lines; the glenoid cavity would have been completely empty, but it was so slightly so that it was easy to confound (in a certain degree) the depression of the deltoid with that which results from a fracture of the surgical neck of the humerus; the elbow would have been brought forwards as far as the costal cartilages, and it was only slightly inclined inwards and forwards; lastly, the fossa infra-spinalis would have been almost entirely occupied by the tumour, on the anterior border of which it, in a measure only, rested."

In a recent case of this accident the same symptoms were observed: by fixing the scapula, raising the arm, and making slight extension, the humerus resumed its proper situation without any difficulty. M. Velpeau concludes that the extreme facility of reduction is an unanswerable argument in favour of the incompleteness of the dislocation. In the third case, a dissection, of what M. Velpeau regards as an incomplete dislocation backwards, was made. The patient injured his shoulder twenty-one years previously to the examination. The author gives the following description of the appearance of the new articulation:

"All the muscles were of large size [the patient was a muscular man], without any indications of either ancient or recent lesions. .......... The head of the humerus occupied all the glenoid cavity, the neck of the scapula, and a small portion of the infra-spinous fossa: it was very much flattened, narrow and thin externally, enlarged internally, and articulated in an almost immoveable manner with the scapula by a capsule, which could only be so called at the internal part of the articulation, where it covered a considerable thickness of the osseous head, for below and externally it was inserted almost at the point of contact between the two bones..........On dividing the capsular ligament the articular surfaces presented the following appearance: The new articular surface of the scapula was much larger than natural, it was formed at the expense of the glenoid cavity and of all the neck of the scapula as far as beneath the coracoid process; hence a complete disappearance of the glenoid prominence was the result. A change in direction indicated very distinctly the situation where this cavity might be divided into two portions; one external, the old normal articular cavity, still a little concave, much lengthened and united by a smooth surface but a little less prominent to the second; the other internal, which was directed to the subscapular fossa, where it terminated by an osseous border. .......... The head of the humerus was not less altered, and instead of presenting a sphere, it was strongly channelled in its centre from above downwards by a deep fissure which divided it into two portions, one external, thin, but rather broad, irregularly convex, corresponding to the old glenoid cavity; the other internal, almost smooth, very thick, in contact with the prolongation of the new articular surface of the scapula."

We have given the description of this dissection in the words of the author, as he believes it confirms incontestably his views on the nature of these dislocations; but so far from agreeing with M. Velpeau we do not understand how this case bears at all on the point in question; it appears to us to be evidently a dissection of a dislocation (probably incomplete) in a direction internal to the glenoid cavity, and must either have been introduced here by mistake, or the palpable absurdity of supporting opinions with such so-called facts would induce us to regard with considerable suspicion the assertions of the author. We do not mean to deny that a partial dislocation in a direction external to the glenoid cavity may take place, but these cases do not appear to us sufficient evidence of its occurrence; and before admitting the existence of such an accident we should wish to hear of its confirmation by other surgeons.

*External and anterior axillary Dislocations.* These form the second class, and M. Velpeau admits these varieties of them: First, sub-pectoral dislocations, where the head of the humerus is lodged between the pectoral and subscapular muscles, the axillary dislocation of authors; secondly, subscapular dislocation, in which the head of the bone rests in the subscapular fossa, but is separated from the hollow of the axilla by the subscapular muscle; and thirdly, subclavicular dislocation, "where the head of the humerus rises towards the root of the coracoid process or the clavicle, and is constricted, as it were, beneath by the superior border of the subscapular muscle."

There is nothing worthy of notice in the remarks of M. Velpeau on axillary or subclavicular dislocations; he considers the former most easily reduced by traction upwards in the direction of the axis of the body, and the latter by the force being directed downwards and inwards.

The subscapular dislocations of M. Velpeau, where the head of the bone is separated from the hollow of the axilla by the subscapular muscle, do not seem to differ, except in name, from those described by Sir Astley Cooper as incomplete dislocations inwards under the coracoid process. M. Velpeau finds these luxations are most easily reduced by the knee in the axilla, the humerus being used as a lever, or else by traction in the horizontal position. The following are the symptoms of these subscapular dislocations as described by the author:

"The pectoralis major is slightly pushed forwards or raised, the hollow of the axilla is nearly effaced, the acromion less prominent, and the deltoid less stretched than in the natural state; the head of the humerus, separated from the skin of the axilla by a thick layer of tissues, is felt with difficulty through the pectoralis major; the fingers can be passed between it and the internal wall of the thorax; the movements of the arm are very limited, and produce a kind of crepitation; the elbow is separated from the thorax and carried forwards; the arm a few lines lengthened, shortened, or of the same length as that of the opposite side."

Crepitation is regarded as the most valuable diagnostic sign of this species of dislocation.

The author enters at great length on the question of the relative frequency of shortening and lengthening of the arm in dislocations; numerous cases are recorded to prove that either symptom may be present, and various methods of measurement are mentioned. M. Velpeau arrives at the conclusion that the arm is more generally lengthened than shortened in luxations of the shoulder; and in cases where shortening occurs the dislocation is usually under the clavicle.

*Varix.* M. Velpeau reviews in detail the different methods that have been used in the treatment of varix, and discards them all in favour of the following operation, which is a modification of that first employed by M. Davat, and which he prefers to all others. M. Velpeau passes a pin, similar to those used in the operation for hare-lip, underneath the varicose vein; around the ends of the pin, which protrude from the integument, a piece of strong waxed silk is firmly twisted, which strangulates that portion of the vein underneath which this pin is placed and the integument above it; the pin is allowed to remain for eight or twelve days, until an eschar is produced. Several of these pins are placed in the course of the varicose vein. This method of treatment must be familiar to many of our readers, as it has been practised in the hospitals of London and elsewhere. We have had opportunities of witnessing the results of many cases treated in this way, and we must confess we consider it far superior to any of the other numerous methods by which it has been attempted to obliterate varicose veins. M. Velpeau operated on a hundred cases without the occurrence of any serious accident; slight erythema and abscess were occasionally the result, and in only one case has this operation in his hands been followed by phlebitis and death. We quite agree with the author in his remark, that no operation will effectually cure varicose veins: the blood is directed into another channel, the smaller veins are distended and become equally distressing to the patient as those which previously existed. There is the same necessity for the support of a bandage or laced stocking after as before the operation. When the pins are allowed to remain under the vein for more than seven or eight days, an ulcer difficult to heal is the result. M. Velpeau aims at producing this, and considers that the vein will not be effectually obliterated without its occurrence. In a great number of cases where we have had an opportunity of observing the effect of this operation, the pins were removed at the end of five days, no ulcer was produced, and the cure was no less effectual. In this chapter a long history of the different operations for this disease will fully inform the reader of their nature and relative merit.

In cases of *varicocele* M. Velpeau recommends the same treatment by the application of the pins and ligature as in common varix. In this operation it is of course very necessary to separate the vas deferens from the veins, and this is best done by seizing the scrotum at its root, separating the vas deferens, which is easily distinguished by its greater hardness, pulling it inwards, and guarding it at the same time with the finger and thumb before passing the needles under the veins. The needles should be applied about the middle of the scrotum, and two are required with an interval of about an inch between them. We have seen the application of this mode of treatment in varicocele, and can recommend it as being equally efficacious and much less severe than many of the operations that are practised for the relief of this distressing complaint.

*The introduction of air into the veins* during surgical operations, though known to all practitioners as occasionally producing most serious consequences, has, from its infrequency, received but little attention from surgical authors, and we have to thank M. Velpeau for having collected together all the known facts on this subject in the present volume. He considers separately what experiments on living animals have taught us; what facts we can collect from observations on the living human subject during the last twenty years, and the value of therapeutical means proposed in similar cases by physiologists and surgeons. It was proved by Bichat and Nysten, that air introduced into the veins in sufficient quantities could cause death. By succeeding experimenters, Barry, Magendie, and Poiseuille, it was found that air would enter of itself in sufficient quantity to produce death in some veins which were opened by any incision; and Poiseuille, in continuing his researches on this subject, discovered that the veins in which this accident could occur were those in which a venous pulsation existed, namely, the subclavians, internal jugulars, and axillary veins. The explanation of this circumstance was given afterwards by M. Berard, who showed that the veins at the root of the neck were so united by cellular and fibrous tissue to the bones in the neighbourhood, that they were prevented collapsing when an opening was made in them, and which remained gaping in the same way as an incision in a perfectly inactive canal would do. M. Velpeau considers that this capability for the introduction of air exists in the internal jugular vein as high up as the larynx. This is not the case in every instance, as we have seen a patient in whom the internal jugular was most freely opened between the thyroid and cricoid cartilages, in his attempts to commit suicide, and yet there were no symptoms to indicate the introduction of any air into the circulation. From the experiments of M. Poiseuille, the introduction of air into the veins of animals is accompanied by a dull sound, though sometimes, especially in horses, by a kind of gurgling. Death usually takes place in from five to fifty minutes, but not instantaneously. The post-mortem appearances exhibit distension of the right auricle and ventricle of the heart, and these cavities contain generally red blood mixed with a great quantity of air, a bloody froth (mousse sanguine). In some cases the same appearances have been found on the left side of the heart, and air has been discovered in the vessels of the brain. M. Velpeau has collected with great industry all the cases that have been hitherto recorded of this accident, about thirty in number. Of those in which death took place a post-mortem examination was only made in seven instances. In these, a quantity of air dilated the right auricle of the heart in some cases, in others the veins only, in one the bloody froth formed in the experiments on animals was present. The sound which accompanies the introduction of air into the veins of man is usually a kind of whistling; and death, when it does take place, is generally instantaneous. From this difference in the symptoms that have been observed between these accidents as occurring in the human subject and produced by experiments on animals, M. Velpeau seems to doubt whether we can with propriety conclude that in these cases death was absolutely produced by the introduction of air into the veins. In our opinion he lays too much stress on the necessary accordance between the result of the experiments on animals and the accident in the human subject; for, though in some of these cases where death took place, the veins opened were the external jugular, the veins of the shoulder, and the subscapular, (which in the experiments recorded were not found capable of admitting air,) yet the symptoms and instantaneous death of the patient could only be accounted for by the occurrence of this accident, and we think the perusal of the cases must satisfy every one that it had occurred. We should be inclined to arrive at a different conclusion from that of M. Velpeau, namely, that air may be taken into the circulation by many smaller veins than was supposed by the experimenters on animals.

The symptoms of this unfortunate accident, when air has been taken into the circulation in large quantities, are, a whistling or gurgling sound, a feeling of faintness, occasionally universal trembling, syncope, and death. When a smaller quantity of air has entered the veins, a feeling of faintness or even perfect syncope may take place, and the patient afterwards recover. Under the head of treatment, M. Velpeau discusses the question, whether preventive measures should be taken in operations at the base of the neck, where wounds of the veins in this situation might occur, these preventive measures consisting of compression of the chest or of the veins between the wound and the heart; and he decides that both these precautions are unnecessary or useless, and that the only resource that remains to the surgeon in these cases is: "1st, to do everything to avoid wounding in operations the internal jugular or subclavian veins; 2d, in cases where it is necessary to penetrate to the neighbourhood of these vessels not to separate the pedicle of the tumour without having compressed it on the side of the heart between the fingers, or surrounded it with a strong ligature; 3, to avoid as much as possible, stretching, pulling, or separating the tissues, elevating the arm or pulling backwards the shoulder or neck when the bistoury approaches the large veins at the summit of the chest." The curative means, or those by which the air may be expelled from the veins of the chest when it has once entered, are very ineffectual. Compression of the chest is perfectly useless, and can have no effect in expelling the air in the human subject, and we have no known remedies by which this accident may be combated.

*Gonorrhœa.* In the treatment of this disease M. Velpeau has tried the effect of iodine, balsam of storax, and gunpowder, each of which remedies has been recommended by some surgeons, and he finds these therapeutical agents useless. He discusses the respective merits of copaiba and cubebs. Patients do not suffer so much from the exhibition of the latter as of the former, and the tone of the stomach is sometimes to a certain degree improved by the cubebs; but the combination of these two medicines in the proportion of one part of copaiba to two of cubebs, produce the most beneficial results. This form of medicine has been recommended by Mr. Liston in the last edition of the Elements of Surgery. M. Velpeau believes that the rheumatism, which has been said to be caused by copaiba is more probably induced by the disease for which it is administered. The rash produced by this medicine is of no consequence. Besides these specific remedies, M. Velpeau recommends in acute cases general bloodletting and leeches to the perineum; and where there is much thickening of the urethra, he has found friction with mercurial ointment of great benefit. The application of small blisters from the anus to the scrotum, as recommended by Mr. Poirson, has been occasionally useful in cases of old standing. In the local treatment of this disease M. Velpeau considers the objection to injections absurd; the abuse only and not the use of these remedies is to be condemned. Sulphate of zinc is, in the opinion of the professor, of no use in recent cases of discharge from the urethra. In gonorrhœa of old standing or in gleets, in very small doses its effects are beneficial; one grain to an ounce of water is the proper proportion. Nitrate of mercury has also been recommended, but is far inferior to nitrate of silver, combined with compression of the urethra. The injection should contain one grain of the salt in every ounce of water, and is used at the same time that the cubebs and copaiba are exhibited internally in the early stages of gonorrhœa. The injection should not be continued longer than three days (about three times a day), lest it produce a fresh irritation in the urethra. This canal should be kept constantly compressed by means of small graduated compresses, dipped in starch, applied from the bulb to the fossa navicularis, and retained by means of a bandage. M. Velpeau's plan of treatment is in all respects one which we should be inclined to recommend, and which we have for some time been in the habit of using, with the exception of the compression of the urethra, of which we have had no experience.

*Artificial anus.* The researches of Dupuytren and his subsequent excellent explanation of the causes, pathology, and treatment of artificial anus would lead us to suppose that this subject would afford but a barren field for fresh research.

"Yet," says M. Velpeau, "in this, as in many other instances, surgery is far from having arrived at perfection. The adherence of the intestine to the neck of the sac is often less intimate than Scarpa supposed. The membranous investment is often wanting. The adhesive inflammation is not always set up on the application of the *enterotome,* and sometimes it is almost impossible not to embrace between the teeth of this instrument a portion of some important organ at the same time with the spur-like process; lastly, artificial anus and fecal fistulæ persist for an indefinite period in spite of the destruction or absence of any spur-like process between the ends of the intestine."

M. Velpeau instances cases of artificial anus in which post-mortem examinations have made good these assertions. In the first case the patient died twelve days after the operation for strangulated hernia. The intestine was found so little adherent to the neck of the sac that the slightest force was sufficient to separate them, and any artificial interference would have been followed most probably by effusion into the abdomen. In the second case the enterotome had been applied, but very little adhesive inflammation had followed its application not sufficient to prevent effusion of fecal matter into the cavity of the abdomen, which was the cause of death. In the third case the infundibuliform space between the two ends of intestine was occupied by a coil of intestine so that the application of the enterotome would inevitably have embraced and opened this portion of bowel as well as the spur-like process itself. M. Velpeau describes these as the chief difficulties the surgeon will have to contend with in the treatment of artificial anus, and they explain the non-success that is met with in many of these cases: moreover fecal fistulæ will persist after the destruction of the spur-like process, or when no such partition between the ends of the intestine ever existed. This may take place in two ways:

"1st. An ulcer commencing in the mucous membrane may cause adhesion of the intestine to the corresponding part of the abdomen; continuing its progress in depth, and having traversed all the tissues this ulcer will cause a fecal abscess which may terminate in a fistula. 2d. If after the reduction of a hernia an opening forms on the convexity of the intestine, the contents may pass out towards the skin and an artificial anus be the consequence. 3d. After the destruction of the spur-like process by the method of Dupuytren, the fecal fistula may resist all attempts to close it."

These observations are supported by numerous cases and dissections, which are too long for insertion here. The treatment of these obstinate cases of fecal fistula demands the best attention and patience of the surgeon. Pressure, styptics, and cauterization will be sufficient to effect a cure in some, but will too often fail. Autoplastic operations have been attempted, but with little success. After a fistula has existed some time the edges become perfectly callous, and the structures in the immediate neighbourhood so changed as to be little inclined to contract adhesions with the new tissue that is placed in contact with them in autoplastic operations. It is also almost impossible to close the opening so effectually as to prevent the escape of a small quantity of fecal matter, which will be quite sufficient to prevent the union of the raw surfaces. The operation M. Velpeau recommends in old cases of artificial anus is that of paring the edges of the opening and bringing them in contact by suture. This operation was effectual in a case, which presented at the hospital of La Pitié, of old-standing artificial anus of the groin, and was performed in the following manner: "The borders of the fistula were first deeply pared so as to form an elliptical opening; four points of suture united the raw edges, and in order to prevent the borders of the wound being too much stretched, semi-elliptical incisions, their concavities towards the wound, were made on either side." We have had an oppôrtunity of witnessing the effect of the autoplastic operation in a case of fecal fistula in the left lumbar region, and though the operation was performed with the greatest care, and every precaution taken to ensure its success, it ultimately failed. We should think, in cases where the artificial opening was but small, the operation recommended by M. Velpeau well worthy of trial.

The article on artificial anus closes the volume before us, and we look forward with pleasure to the perusal of the additional part of this work promised by the author in his preface, and which (from the large portion of the present volume that is occupied by articles on diseases of the eye) will probably be devoted more especially to subjects of general surgery. The publication of these clinical lectures cannot fail to add considerably to the fame M. Velpeau has already acquired for erudition and accurate observation.

---

